# The effect of ultrasound on the crystallization-precipitation process of transforming sodium amoxicillin into amoxicillin trihydrate^☆^

**DOI:** 10.1016/j.ultsonch.2025.107590

**Published:** 2025-09-26

**Authors:** Aicha Ladaidi, Loïc Hallez, Isabelle Pochard, Nicolas Rouge, Jean-Yves Hihn

**Affiliations:** a Université Djilali Bounaama Khemis Miliana, Rue Thniet El Had, Khemis Miliana, Algeria; b Université Marie et Louis Pasteur, CNRS Institut UTINAM UMR 6213 30 avenue de l’Observatoire, 25009 Besançon cedex, France

**Keywords:** Amoxicillin, Sonocrystallization, Crystal morphology, Ultrasound, Pharmaceutical

## Abstract

Crystallization is a process used in many industrial fields. However, in the pharmaceutical industry it plays a crucial role as the quality of the drug (finished product) in terms of therapeutic efficacy and stability is strongly related to the physical properties of the active ingredient, as well as other excipients determined and controlled through this process. This paper deals with the feasibility of the transformation of amoxicillin sodium salt into amoxicillin trihydrate. It will also be the opportunity of studying the physical properties of the crystals obtained (size and size distribution, shape, purity, morphology) as a result from the interaction of the crystallization-precipitation process with or without ultrasound and the selected antibiotic amoxicillin. The limiting factor for sonocrystallization is pH, the optimum pH being 4.5. Indeed, for a pH below 2, the crystallization yield does not exceed 10 %. Ultrasound has a synergistic effect on crystallization by improving the yield and the fineness of the powder, as well as on the kinetics of the reaction in relation to the nucleation time, even if it is not necessarily possible to attribute this phenomenon to solubility or de-supersaturation. For 30 min experiments, the best yield of 95 % was obtained using a low frequency (20 kHz), compared to the yield obtained without ultrasound (69 %) or with higher frequencies (581 kHz – 72 % and 864 kHz – 65 %). In addition to the ultrasound frequency used, temperature, pH and sonication time also have a profound effect on the product’s crystal morphology and size. In the case of low frequency, particle size ranged from 0.4 to 60 μm, and from 0.7 to 250 μm under silent conditions.

## 1 Introduction

Crystallization is a fundamental process which occurs when supersaturation is reached [[1], [2], [3], [4], [5]]. It consists in two steps in which, first, the crystals will appear, before growing during the second step crystal growth. Since the relative dominance of nucleation and growth may change depending on the experimental condition [6], several parameters are relevant. The method used to obtain supersaturation depends on the characteristics of the crystallization system: some solutes crystallize easily from the solution once cooled, such as Insulin, Urease and Trypsin [7], while others can only be obtained after removing some of the solvent (by evaporation) which is the general case for salts [8]. In addition, Acetaminophen [9] or Adipic acid [10] does not crystallize during cooling, but during a subsequent reheating. While this process is generally used for metallic salts, it is less frequently used for pharmaceutical compounds due to possible degradation by heating. For other solutes, the addition of another substance to the system leads to changes in equilibrium conditions and precipitations. Supersaturation is sometimes obtained as a result of a chemical reaction between two or more substances, and one of the reaction products is precipitated [11]. Thus, high quality products may be obtained by crystallization in terms of therapeutic efficiency and stability and with controlled physicochemical properties such as particle size distribution, shape and morphology. It has been reported that more than 90 % of pharmaceutical products contain an active ingredient in crystalline form [12]. Nevertheless, the crystallization process is quite slow, which can be a disadvantage on an industrial scale. Powerful ultrasound can reduce nucleation times by one to three orders of magnitude for production of several organic or inorganic crystals. The precise mechanism of this phenomenon is not yet clear [13], but various theoretical explanations involve the action of inertial cavitation bubbles: cooling effect, pressure effect, segregation effect and evaporation effect. All these effects appear possible and may in fact act in a complementary way [14,15]. Sonocrystallization has been used in pharmaceutical applications, for example to enhance crystalline product quality of Paracetamol. Requirements of the specifications relate to purity, particle size, and size distribution [16]. This problem of size and shape of the crystals, modulable by ultrasound action, is a major concern for therapeutic effects [17].

**Fig. 1:**
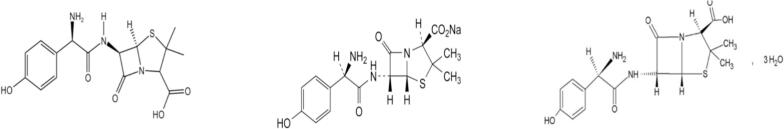
Structures of amoxicillin (left), amoxicillin salt (center) and amoxicillin trihydrate (right).

In our study, the target organic molecule is one of several semi-synthetic derivatives of 6-aminopenicillanic acid (6-APA) developed in Beecham, England in the 1960s [18], and is available in pharmaceutical preparations such as salt, amoxicillin sodium and also as hydrates as shown in Fig. 1. Amoxicillin sodium is highly hygroscopic, while trihydrate is not hygroscopic. Among the free bases, sodium salt and trihydrate, amoxycillin trihydrate is the most stable solid form [19].

Amoxicillin trihydrate and its salts have been monographed in several pharmacopoeias such as the International Pharmacopoeia, the British Pharmacopoeia, and the US Pharmacopeia. [[20], [21], [22]]. Hydrated forms of amoxicillin such as monohydrate, dihydrate and trihydrate are available. Among the hydrated forms, trihydrate is the most stable [23]. Gibbs’ free energy analysis at 100 °C, both in ambient air and at 75 % relative humidity, showed that amoxicillin trihydrate is more stable than the anhydrous form under high temperature and humidity conditions [24]. Regarding the impacts of ultrasound on crystallization of amoxicillin trihydrate, emphasis is placed on induction time, crystallization yield, shape, morphology, crystal size and distribution size compared to the conventional method, because a main issue in the production process is the recovery of the molecules in a solid state. D. Awari et al. studied the crystallization of ampicillin trihydrate under the influence of ultrasound, which proved to be an effective way to obtain smaller crystals (89.77–3.88 μm) with improved bioavailability [25]. Regarding amoxicillin, several researchers [[24], [25], [26], [27], [28]] have studied its purification, with studies focused on the effect of critical acid as a crystallizing and purifying agent on a mixture containing amoxicillin trihydrate and 4-hydroxyphenylglycine. It was determined that the average particle diameter of pure amoxicillin trihydrate was between 1250 and 1500 µm, under pH 5.5 conditions [26,27]. Amoxicillin trihydrate samples recrystallized from aqueous solutions at pH 7 and pH 9 appear to have superior physicochemical properties [28]. The results of these studies show that the crystallization technique can change the crystal shape and particle size, depending on the type of crystallization medium and the crystallization pH [29] or temperature [30].

**Fig. 2:**
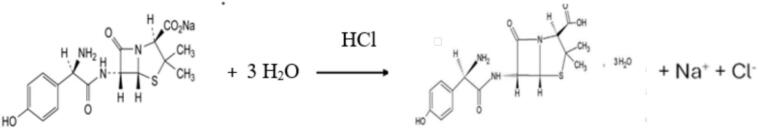
Scheme of the process for transforming amoxicillin sodium salt into amoxicillin trihydrate.

In the present study, the main objective was to verify the feasibility of the transformation of sodium amoxicillin into amoxicillin trihydrate and to optimize the operating conditions of the process. The methodology followed to carry out this study is a 2-step process. The first step consists in optimization of operating conditions of the crystallization process by studying the influence of temperature, concentration and nature of the chosen acid on the precipitation of amoxicillin trihydrate. Indeed, crystallization of amoxicillin trihydrate is performed by pH adjustment. The process is based on a crystallization by precipitation, induced by a chemical reaction (Fig. 2), based on the displacement of the solubility of the final product with change in pH, by addition of acid.

Once these conditions are met, a second step focuses on the application of low and high frequency ultrasound during synthesis to evaluate the effect of ultrasound on the quality of the products obtained (morphology, size distribution and crystallization yield). Different analytical methods cited in the section above were used, but as our target molecule is an antibiotic, it is necessary to check the therapeutic efficacy of the different crystallized products by using the disk diffusion technique. Then, for the first time, it was possible to highlight the added value of the application of ultrasound in terms of yield, quality of the crystals formed (expressed in size, shape, and size distribution of the particles obtained), and on its therapeutic efficacy.

## 2 Materials and methods

### 2.1 Materials

Amoxicillin trihydrate (used as the reference substance with 94.86 % of purity) and amoxicillin sodium salt with 89.2 % of purity were kindly provided by the Saidal Pharmaceutical Group Company (Algeria). Distilled water was used as solvent. Different acids purchased from Sigma-Aldrich were used as precipitating agents, such as hydrochloric acid (37 %), sulfuric acid (98 %), phosphoric acid (85 %), and nitric acid HNO_3_ (65 %). All chemicals were used as received.

### 2.2 Analytical methods

To characterize and identify the crystallized products resulting from the different amoxicillin trihydrate tests, several techniques were used such as Attenuated Total Reflection Fourier Transform Infrared (ATR-FTIR) spectroscopy measurements. Spectra were collected using a Shimadzu Iraffinity-1 spectrometer, directly on the powders with a measurement range from 4000 to 550 cm^−1^ and a resolution of 1 cm^−1^. UV–Visible spectrophotometry measurements were conducted using a UV-line 9400 Secomam spectrophotometer, with a maximum absorbance at 272 nm. Metrohm 831 was used for Karl Fischer titration to determine moisture for both high water content and amoxicillin trihydrate crystallized samples. The pH-meter is a Mettler-Toledo Sevencompact. Powder X-Ray diffraction measurements were taken with a Bruker D8 Advance x-ray diffractometer equipped with a Lynxeye detector. Spectra were collected using a copper kα anode (λ = 0.1542 nm) operating at 40 kV. Diffraction patterns were collected at 25 °C and over an angular range of 5°–70° or 100°, 0.02° with a step rate of 0.3 s/step. SEM is a Tescan MIRA3 FEG (Field Effect Gun) SEM. Morphological images were obtained at low voltage (5 keV) and low current (62pA) using secondary electron detectors in the chamber (SE) and in the objective lens (In Lens). The SEM pictures presented here are always representative of several investigations all around the sample surfaces.

### 2.3 Crystallization protocols

All crystallization tests were carried out in a 500 mL double-walled reactor (80 mm diameter) except for the high frequency test (55 mm diameter) due to transducer adaptation constraints. They were filled with 250 mL of solution, and equipped with a cryothermostat bath (Julabo) to control the temperature. Crystallization apparition times were detected by the formation of turbidity visible to the naked eye and verified twice on a video recorded by a camera.

#### 2.3.1 In silent conditions

A fixed amount of amoxicillin sodium salt (10 g) is added in 250 mL water to be close to the supersaturation conditions (0.04 g/mL, leading to an initial pH = 9.4 ± 0.05). Then, fixed volumes of acids under a 100 rpm agitation are added. Indeed, preliminary tests at different rotation rates around these values does not induce any change in crystallization, so that 100 rpm has been chosen as reference. Addition of various volumes and acid natures leads to a final pH which is systematically monitored, since it is directly related to amoxicillin trihydrate solubility.

After precipitation of the amoxicillin trihydrate, filtering and drying were performed (either in an oven at 60 °C or under vacuum) to obtain the amoxicillin trihydrate in powdered form. Crystallization yield is calculated by the weighing method using the following formula:

(1) $$\text{Yield of crystallization}=\frac{\text{Mass of crystals of amoxicillin trihydrate collected at the end of the precipitation}}{\text{Mass of crystals of amoxicillin sodium salt intially dissolved in that volume}}*100$$

#### 2.3.2 Under sonochemical conditions

**Fig. 3:**
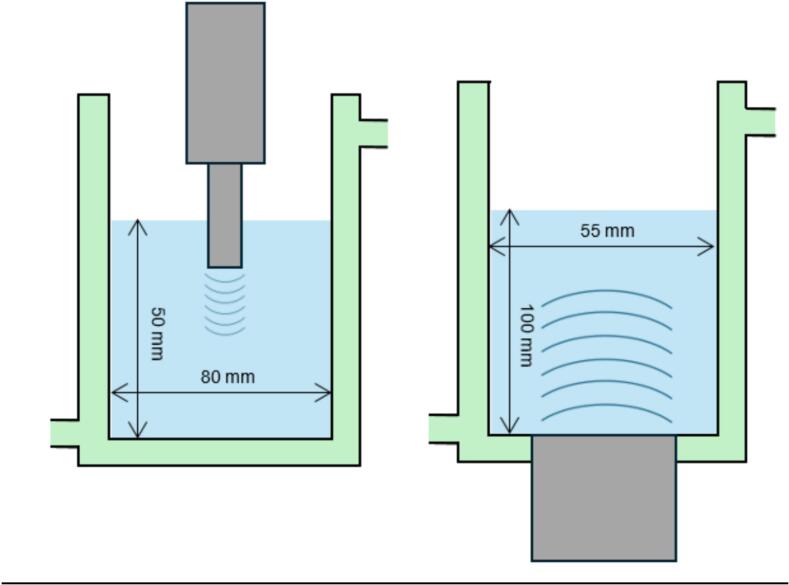
Scheme of reactors used in sono-crystallization experiments of amoxicillin trihydrate − Low frequency sonoreactor on the left (20 kHz) − High frequency sonoreactor (581 and 864 kHz) on the right.

Low frequency ultrasound is generated by the 20 kHz (wavelength λ_20kHz_ = 7.4 cm in water) Sinaptec's NextGen ultrasound system with a horn diameter of 25 mm independent from the reactor and placed at the top (Fig. 3 left – horn at the reactor middle). High frequency is generated by a planar multi-frequency transducer E/805/T 55 of 55 mm diameter from Meinhardt Ultraschalltechnik fixed at the reactor bottom (Fig. 3 right). Two high frequencies are available (581 and 864 kHz – respective wavelengths λ_581kHz_ = 0.255 and λ_864kHz_ = 0.171 cm in water), with 55 mm of diameter placed at the bottom of the vessel. As the reaction rate is linked to the acoustic intensity transmitted to the reactional volume, electrical powers were tuned to adjust the transmitted power (measured by calorimetry) at two values: 30 W.L^−1^ and 120 W.L^−1^, irrespective of frequency. Therefore, prior to the crystallization tests, calorimetric measurements were conducted with deionized water up to reach these values for both frequency systems (described in Fig. 3). The experiments carried out allowed us to highlight the influence of frequency (20 kHz and 581 kHz) and sonication time (10 min or 30 min), not only on crystallization yield but also on the quality of the final crystallized product expressed in terms of particle size, shape and distribution size. It is important to note that ultrasound was started before the addition of acid, so as to remain always under the same conditions.

### 2.4 Evaluation of antibacterial activity of the obtained amoxicillin trihydrate

The antibacterial activity of crystallized amoxicillin trihydrate, produced with or without ultrasound was measured by the disk diffusion technique. Five types of pathogenic bacteria, including two G-negative bacteria (*Escherichia coli and Pseudomonas aeruginosa*) and three G-positive bacteria (*Staphylococcus aureus, Staphylococcus epidermidis and Bacillus subtilis*) were tested. For the disk diffusion method, Mueller-Hinton agar was used. Sterile paper disks (9 mm) were impregnated with an amoxicillin trihydrate solution (0.1 mg/mL). Antibiotic disks were then placed on the surface of the agar using a dispenser that dispenses multiple disks at the correct distance apart. The plates were inverted and incubated for 24 h at 37 °C. The tests were repeated three times. The inhibition ring diameter of growth, which appeared around the disks, was measured.

## 3 Results and discussion

### 3.1 Crystallization of amoxicillin trihydrate in silent conditions

**Fig. 4:**
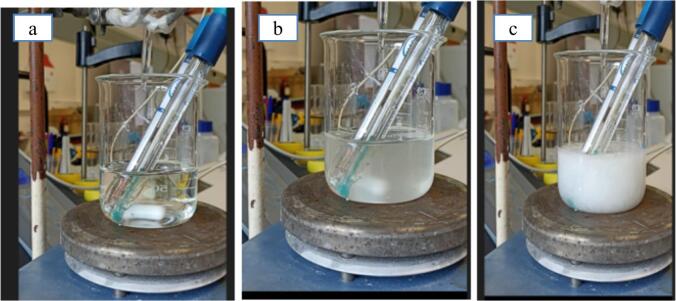
A) supersaturation (0 s) b) Nucleation (28 s) c) Final crystal product (80 s).

First, crystal growth is detected visually by noticing turbidity. It is interesting to note that, for all tests carried out in silent conditions as shown in Fig. 4, the time lag before the appearance of the first crystals is observed between 20 and 32 s (depending on temperature), indicating a rapid nucleation compared to values reported in other works on crystallization, where the appearance of the first crystals can take minutes or even hours. For example, N. Lyczko et al. mentioned a time lag of 9000 s (150 min) in silent conditions (200 mL with a stirring of 500 rpm) for the appearance of the first crystals of K_2_SO_4_ (inorganic substance) at an absolute supersaturation of 0.0156 g K_2_SO_4_/g of water. This can be considerably reduced to 1000 s (16 min**)** in 20 kHz–20 W ultrasound presence [31]. In the case of an organic pharmaceutical molecule paracetamol, Bhangu, S.K et al. reported that the use of ultrasound led to a reduction in induction time, from 360 in the case of conventional crystallization (antisolvent crystallization) to only 30 s under the effect of 10 W ultrasound in 200 mL solution [32]. In our conditions, induction time is about 25 ± 6 s.

This confirms that these time lags are highly dependent on the chosen technique, i.e. the molecules in question and the crystallization medium.

Then, in order to determine the optimal conditions i.e. the best amoxicillin trihydrate crystallization yield, a series of experiments were carried out while varying i) the acid nature and its addition volume (Table 1) ii) the final pH of the reaction solution (Table 2) iii) the concentration/volume acid ratio (Table 3) iv) the temperature (Table 4). All tests have been repeated at least three times, and only the average value was kept in the tables.

**Table 1:** Influence of the nature of acid added as precipitating agent on crystallization yield of amoxicillin trihydrate (Final pH 4.5–––100 rpm − 22 °C − silent).

**Acid nature (1 N)**	**HCl**	**H_2_SO_4_**	**H_3_PO_4_**	**HNO_3_**
C**rystallization yield (%)**	**52.66 % ± 1.35**	**35.89 % ± 2.04**	**48.66 % ± 1.05**	**47.30 % ± 1.57**
**Acid volume (mL)**	**50**	**90**	**110**	**70**

**Table 2:** Variation in crystallization yield depending on the pH of precipitation, 7 °C silent conditions, 4 N hydrochloric acid solution.

**pH of precipitation**	**Acid volume (mL)**	**Crystallization yield %**	**Moisture content %**
**1.98**	**9.4**	**10.60 ± 2.17**	**13.22**
**2.98**	**9**	**42.40 ± 1.76**	**13.25**
**4.5**	**7**	**67.27 ± 0.15**	**13.62**
**7.7**	**3.5**	**57.86 ± 1.04**	**12.54**

**Table 3:** Variation in crystallization yield depending on the volume of hydrochloric acid added, silent.

**Acid volume (mL)**	**HCl Normality (N)**	**Crystallization yield %**	**Identification by IR**	**Identification by Iodometric Dosage**	**Moisture content %**
**50**	**1**	**52.66 ± 0.71**	**+**	**954.23**	**13.45**
**32**	**2**	**55.78 ± 1.26**	**+**	**988.63**	**12.15**
**17**	**3**	**59.48 ± 2.05**	**+**	**984.54**	**13.60**
**9.5**	**3.7**	**64.35 ± 1.78**	**+**	**1023.35**	**12.24**
**7**	**4**	**67.27 ± 1.12**	**+**	**1026.44**	**13.62**
**4.8**	**5**	**58.23 ± 1.08**	**+**	**1004.24**	**12.48**
**2.1**	**6**	**51.17 ± 1.33**	**+**	**966.46**	**13.52**

**Table 4:** Variation in crystallization yield depending on precipitation temperature, 20 min silent by adjusting pH to 4.5 by hydrochloric acid 4 N addition.

**Temperature (C°)**	**Crystallization yield %**	**Identification by IR**	**Identification by Iodometric Dosage**	**Moisture content %**
**07**	**67.40 ± 1.02**	**+**	**1030.78**	**13.25**
**10**	**66.66 ± 0.95**	**+**	**1002.30**	**13.62**
**15**	**65.35 ± 0.55**	**+**	**992.32**	**12.54**
**22**	**64.10 ± 1.03**	**+**	**965.35**	**13.02**

First, various amounts of hydrochloric, sulfuric, phosphoric and nitric acids were added to the amoxicillin sodium salt solution at room temperature under 100 rpm. Volumes were adjusted to reach a final pH value of 4.5 in all cases. This is the optimal value for maximizing trihydrate precipitation according to the curve of solubility of the target molecule at 37 °C adjusted with hydrochloric acid or potassium hydroxide solution [33], knowing that precipitation begins well before this pH. In the pH range between 4 and 6, amoxicillin trihydrate shows a minimum solubility of about 1.25 10^−2^ mol/L.

It can be seen that, at a given pH value, the best results i.e. the highest crystallization yields, were obtained with hydrochloric acid. Therefore, this acid was selected for the rest of the study. Even if the optimal pH value for maximizing trihydrate precipitation is known to be about 4.5, various final pH values reached by adding various hydrochloric acid volumes were tested, to observe its influence on crystallization yields and powder moisture content (after drying at 60 °C).

As shown in Table 2, a small variation in added acid volume results in significant changes in pH and consequently in crystal formation and moisture content. It is noteworthy that a slightly acidic pH (4.5) gives a crystallization yield of 67.3 %, well above those obtained with more alkaline or more acidic pH. Below pH = 2, the crystallization yield drops significantly. It was found that below the optimal pH, a small quantity of around 0.2–0.3 mL causes a notable change in the pH of the medium as amoxicillin trihydrate has an acidic character. We bear in mind that the pH measured for a concentration of 0.05 mg/mL is 4.63 and that, therefore, in a more acidic environment, it is the reverse process which occurs, that is to say the dissolution of amoxicillin trihydrate instead of its precipitation

As demonstrated in Table 3, the concentration of the precipitating acid also impacted the precipitation of amoxicillin trihydrate in terms of volume required for complete precipitation and, subsequently, the crystallization yield.

Finally, the best conditions from above (an optimal concentration of 4 N hydrochloric acid under an average stirring speed of around 100 rpm) were implemented at different temperatures (Table 4).

The results obtained showed that amoxicillin crystallized more at low temperatures (7 °C) with an optimum yield of around 67.4 %. We have been able to conclude from previous experiments is that a maximum 69 % of crystallization of amoxicillin trihydrate is obtained by using hydrochloric acid as a precipitating agent after 30 min. This is the best acid, leading to a significant change in solubility and with an optimal concentration of 4 N under an average stirring speed of around 100 rpm and working at a temperature of 7 °C, followed by drying at 60 °C. These operating conditions are applied in the following section to draw conclusions regarding the added value resulting from application of ultrasound on the crystallization process.

**Fig. 5:**
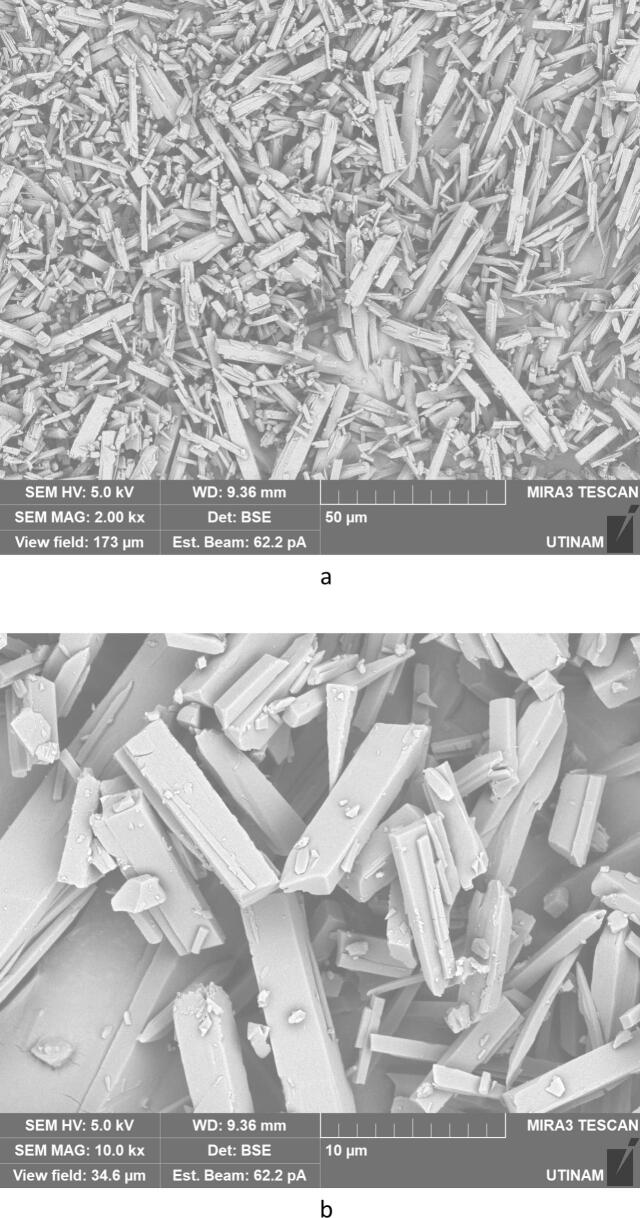

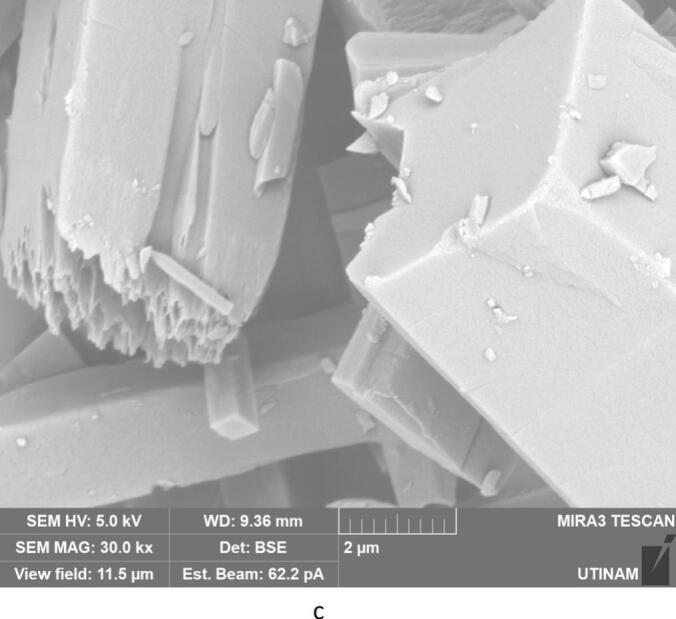
SEM analysis with three magnifications, 2.00 kx – 5a, 10.00 kx 5-b, and 30.00 kx − 5c, respectively, of crystals obtained at 7 °C (effect of temperature on the morphology of amoxicillin trihydrate particles). These tests were carried out under the same operating conditions; same initial concentration 0.040 g/mL in sodium amoxicillin and at a final pH of 4.5 with stirring at 100 rpm.

**Fig. 6:**
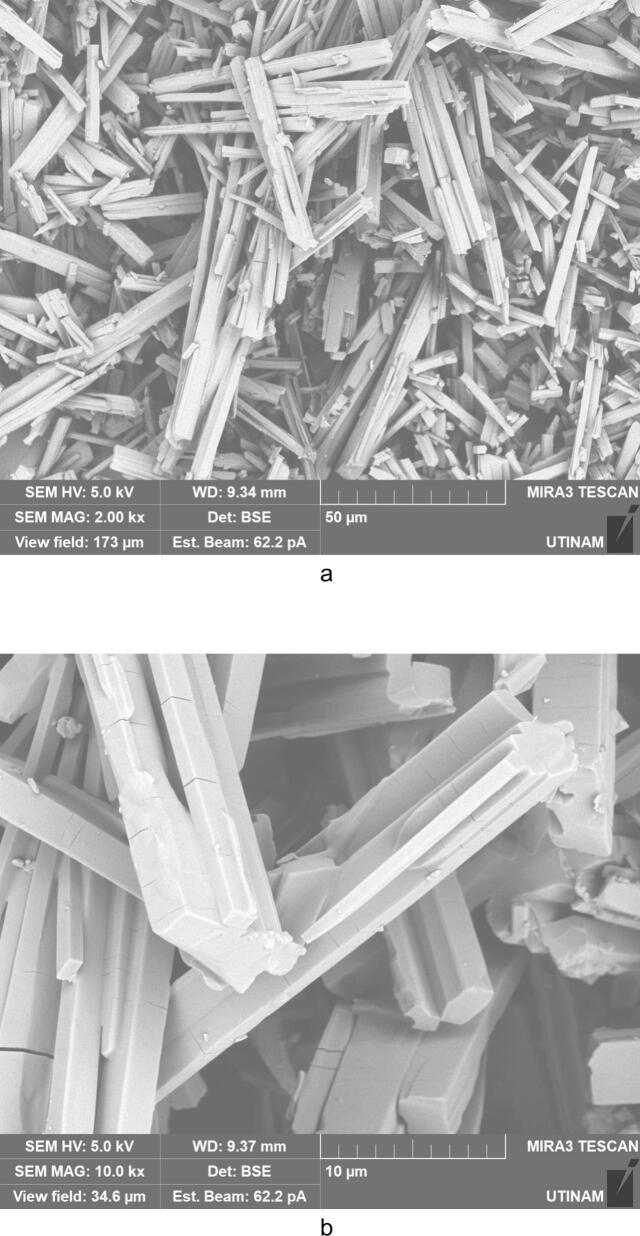

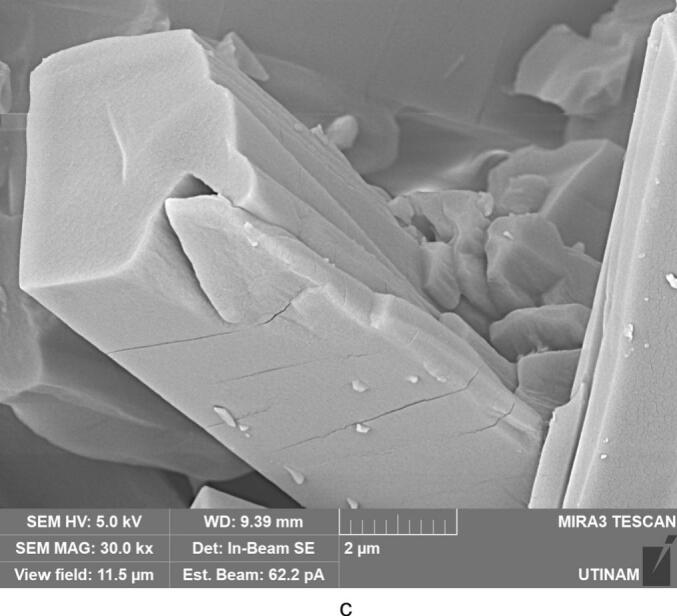
SEM analysis of crystals obtained with three magnifications, 2.00 kx − 6a, 10.00 kx – 6b and 30.00 kx – 6c respectively, in silent conditions at 22 °C (effect of the temperature on the size of amoxicillin trihydrate particles). These tests were carried out under the same operating conditions; same initial concentration 0.040 g/ml in sodium amoxicillin and at a final pH of 4.5 with stirring at 100 rpm.

By performing crystallization tests under silent conditions at different temperatures, we found that temperature lead to changes, not only on the crystallization yield as summarized in Table 4, but also on the morphology and on the size of the crystallized amoxicillin trihydrate, as illustrated in Fig. 5, Fig. 6, with SEM pictures obtained at 7 °C and 22 °C, respectively. In both cases it can be clearly seen that amoxicillin crystallized in the form of needles in different sizes (using 2.00 kx magnification). When examining the crystallized particles under a magnification of 10.0 kx, the particles appeared in cuboid shape with a square or rectangular base. By further enlarging the SEM images at magnitude of 30.0 kx (Fig. 5), the resulting crystals showed that one of the ends was naturally porous, while at 22 °C the formation of interlayers was identified. Concerning size, large particles were observed at this temperature with widths varying between 1.8 and 6.0 μm and lengths between 7 and 65 μm. At 7 °C, smaller particles were formed with widths ranging from 0.5 to 2.46 μm and lengths from 3.5 to 12 μm. These results were similar to those observed in the work of Kakran et al. [34] and C. Li et al. [35].

This variety in size is probably due to the solubility of the molecule in question, which depends on temperature. Usually at low temperatures, diffusion and growth kinetics decrease at the interface of the crystal boundary layer leading to the formation of small particles.

### 3.2 Crystallization of amoxicillin trihydrate under ultrasound conditions

#### 3.2.1 Effect of ultrasound frequency on crystallization yield and morphology

**Fig. 7:**
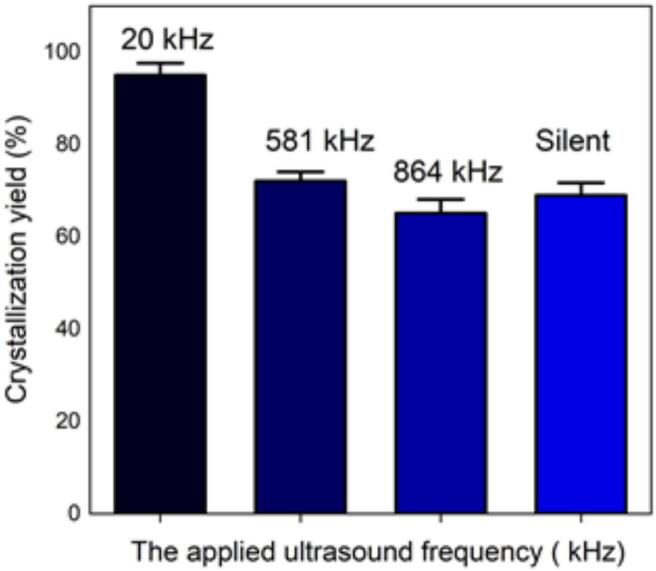
Crystallization yield of amoxicillin trihydrate under 30 min sonication at different frequencies and 120 W/L compared to silent conditions.

**Fig. 8:**
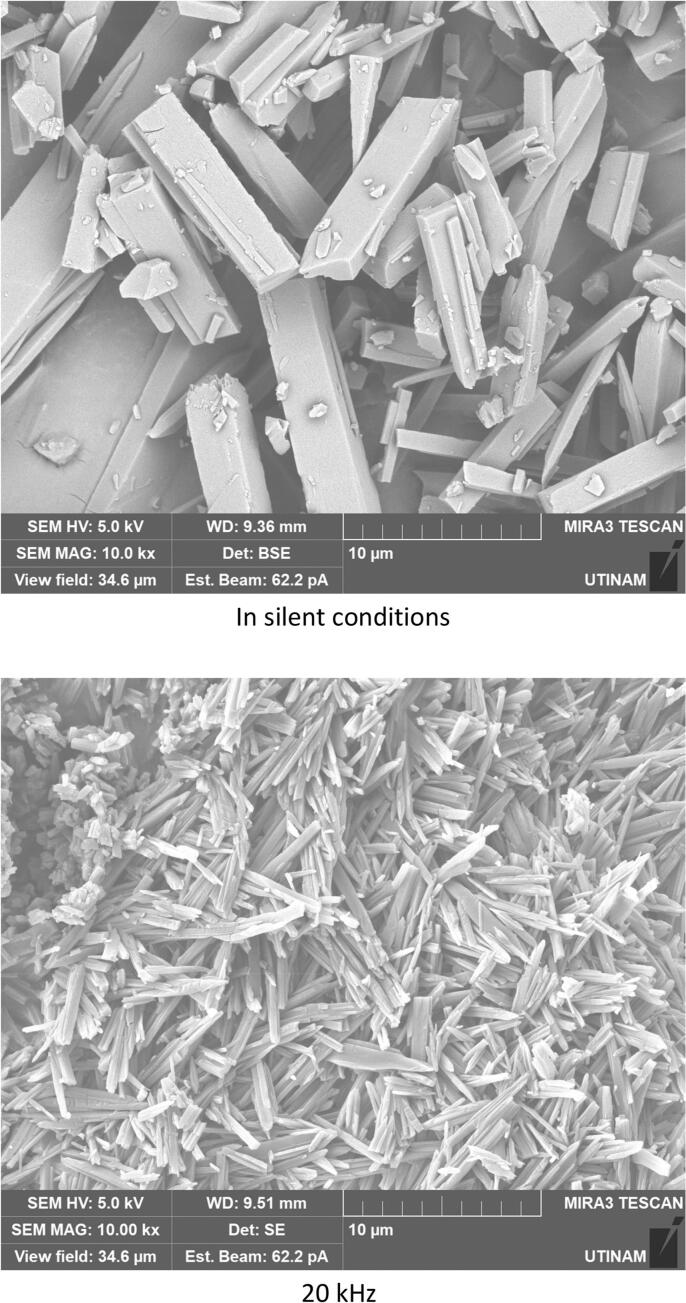

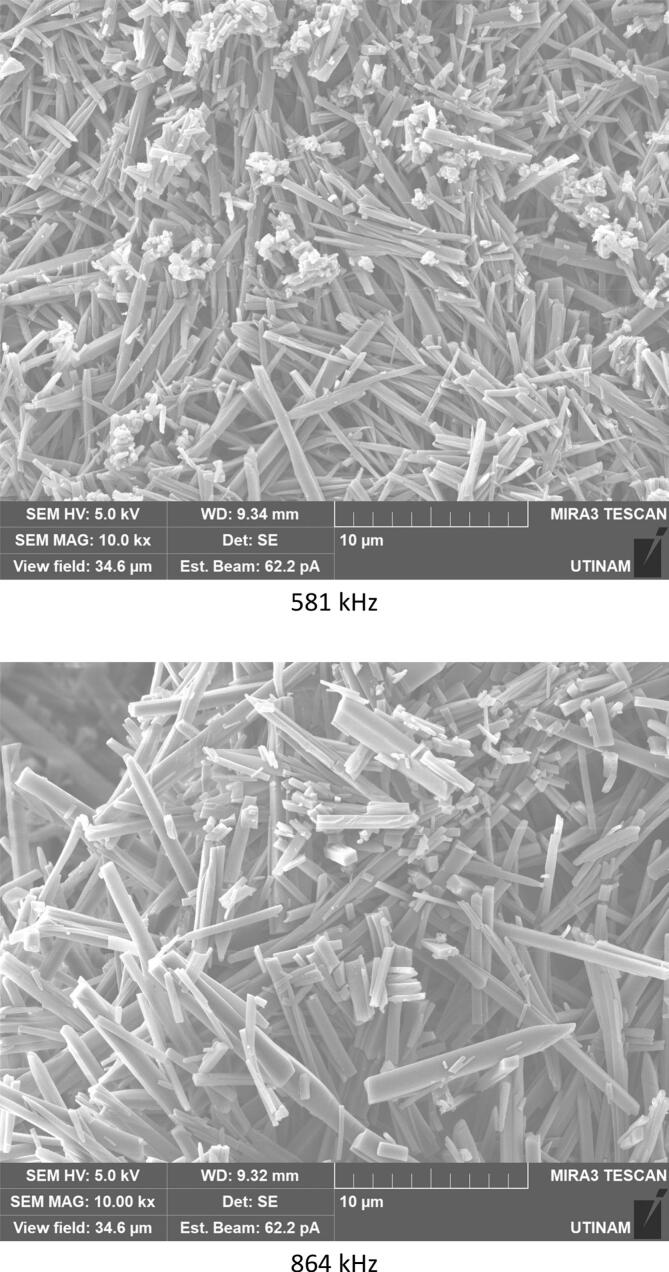
SEM analysis of amoxicillin trihydrate crystals under 30 min sonication at different frequencies and 120 W/L compared to silent condition − magnification 10.0 kx.

The effect of ultrasound on crystallization of our molecule target was investigated by using three frequencies (20 kHz, 581 kHz and 864 kHz). It must be noted that the transmitted power measured by calorimetry was always fixed at 29.5 W by adjusting the electrical input. This value ensure the presence of cavitation for all frequencies, but no chemical dosimetry measurement were conduct because of their lack in reliability in such media (no correspondence between pure water and solution with particles apparition and growth) [36]. The results obtained and displayed in Fig. 7, Fig. 8 indicate that the ultrasound has a significant effect on the yield, size, and shape of the particles formed and crystallized compared to the particles obtained in silent conditions. Due to the significant heat generation by ultrasound, temperature values immediately stabilize at a higher temperature range always around 30–34 °C even if a cooling system was used. Despite the optimal temperature previously found around 7 °C, it was been decided to maintain this temperature range which is closer to real processing conditions, without unreasonable cooling energy consumption.

Fig. 7 clearly shows that crystallization yield is inversely proportional to ultrasound frequency: low frequency leads to yield varying between 92 % and 95 % compared to results obtained at 581 or 864 kHz (∼70 %). Beside the effect of ultrasound on the crystallization yield, particles shape and size were also influenced, it can be clearly seen from comparing SEM images of amoxicillin (Fig. 8). In silent conditions, amoxicillin trihydrate crystallized in orthorhombic form and with different sizes ranging from 1.25 to 2.70 μm in agreement with prior studies by Boles et al. [37] for comparable compounds. For particles crystallized under the effect of low or high frequency ultrasound, two morphologies were observed. The majority of these particles are crystallized in the form of striated stalks, composed of jointing elements ending in a needle shape and therefore with surface porosity between the elements. Others, representing a minority, kept the same morphology (orthorhombic) obtained by conventional crystallization and with different sizes. The presence of the second morphology may mean that not all formed particles were influenced by ultrasonic irradiation in the same way. In the case of high frequency (581 kHz) particle widths varied from 0.37 to 0.58 μm.

In relation to particle size, crystallized particles have a proportional tendency related to the ultrasound frequency applied: the smallest particles (with an average size of 0.24 µm in width) were obtained using low frequency (20 kHz). This can be attributed to the higher role of cavitation at low frequency over acoustic streaming which is the dominant effect at high frequencies. The appearance of small particles is the result of fragmentation caused by the cavitation phenomenon. This effect is more pronounced at lower frequencies. In a study related to the sonocrystallization of an active pharmaceutical ingredient carried out within the framework of the development of a candidate molecule it was reported that the use of ultrasound in a batch mode with an ultrasonic probe operating at 20 kHz frequency with an effective energy input of 100–200 W for 10 g of the material has a noticeable effect on the particle size. In the presence of ultrasound irradiation at frequencies and transmitted powers of the same range of magnitude than used for this study, particle size decreased from 100–200 µm to 20 µm [37]. In the light of these results, the low frequency of 20 kHz is considered as the optimal frequency leading to the smallest particles. The same results have been reported by many researchers who found that low frequencies between 20 and 100 kHz can generally be considered as optimal for improving nucleation and fragmentation of many studied molecules [32,38,39,40]. Since our objective was to study the effect of high and low frequencies on the crystallization of our target molecules, only 581 kHz as high frequency, 20 kHz as low frequency and the silent mode were kept as entry parameters for the following part of the study.

#### 3.2.2 Effect of ultrasound frequency on amoxicillin trihydrate characteristics

**Fig. 9:**
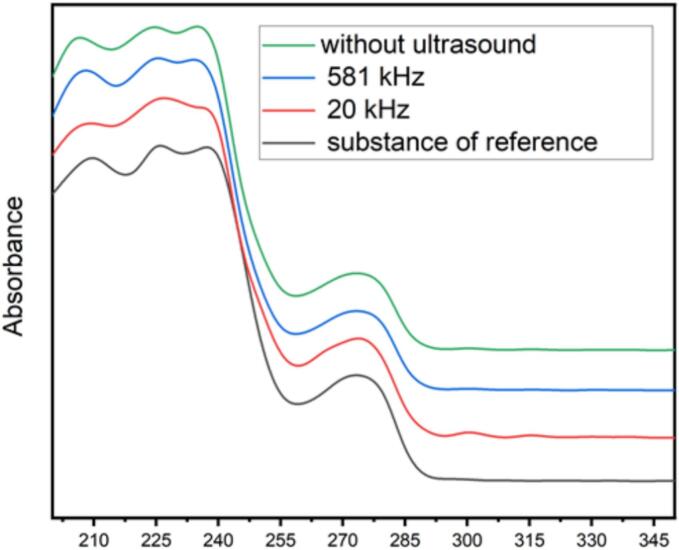
UV/Vis spectra analysis of amoxicillin trihydrate obtained in silent and under ultrasonic conditions compared to a reference substance.

**Fig. 10:**
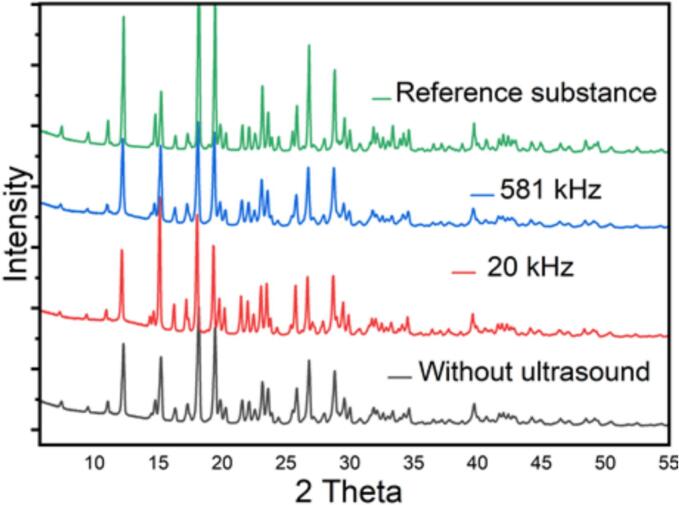
XRD diffractogram analysis of amoxicillin trihydrate obtained in silent and under ultrasonic conditions compared to a reference substance.

**Fig. 11:**
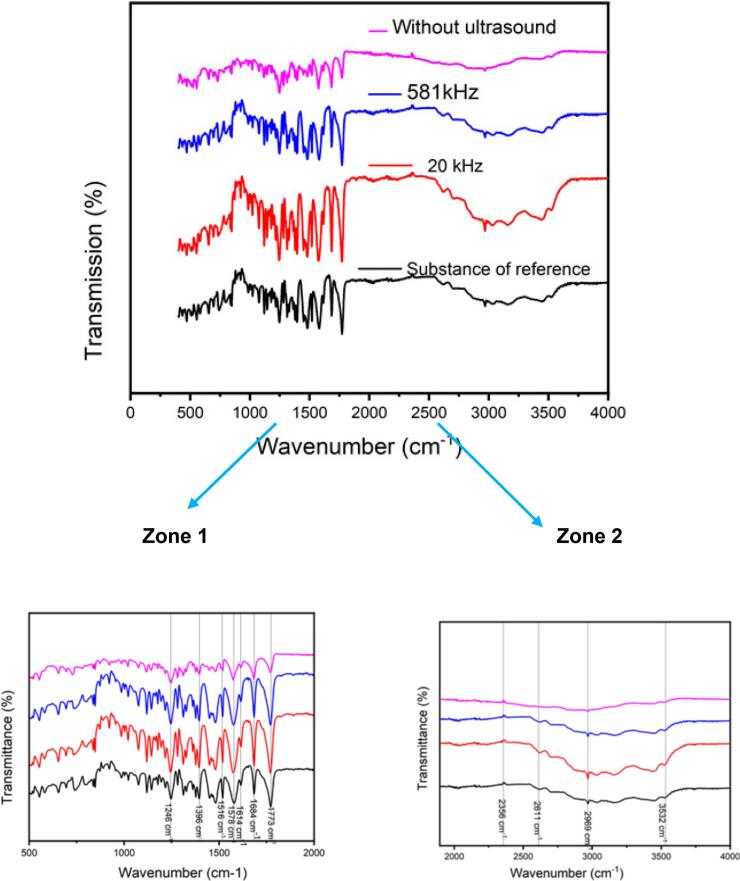
ATR-FTIR analysis of amoxicillin trihydrate obtained in silent and under ultrasonic conditions compared to a reference substance with a close up of the regions between 500 and 2000 and 2500 – 4000 cm^−1^ in high resolution.

All products from the different experiments were analyzed and compared to an internal reference standard. The analysis carried out includes identification by Infrared, UV-spectrophotometry, X-ray diffraction, and SEM as shown in the following Fig. 9, Fig. 10, Fig. 11.

Analysis of the Figures illustrating the identification of amoxicillin using different techniques allowed us to draw the following conclusions:

✓ The different aqueous solutions of amoxicillin trihydrate crystallized under different treatments exhibit an absorbance maximum located at 272 ± 2 nm.
✓ The XRD diffractograms of all analyzed samples show the same profile with a slight change in intensity compared to the standard.
✓ The analysis of FTIR spectra of crystallized amoxicillin trihydrate samples allows a division into two regions:

##### 3.2.2.1 Region 1 from 500 to 2000 cm^−1^

Analysis of all absorption bands of all ATR-FTIR spectra of crystallized amoxicillin trihydrate samples, as well as the reference substance reveals that all the spectra of these corresponding samples are identical. The characteristic peaks of the different functional groups of amoxicillin that could be determined are as follows: a characteristic peak of the group (C=O) (betalactam) observed at 1773 cm^−1^ another band (C=O) amide at 1682 cm^−1^, the characteristic peak of the aromatic C=C situated at 1614 cm^−1^, as for the group COO^−^ at 1578 cm^−1^, the group NH is located at 1516 cm^−1^, another peak appeared at 1396 cm^−1^ due to the stretch of (COO^−^) and phenol (–OH). At 1246 cm^−1^ characteristic of phenol CO and amide as well as NH bend CN was detected. These results are in good agreement with those reported by C. B. Boutelliez et al. [41].

##### 3.2.2.2 Region 2 from 2550 to 4000 cm^−1^

The same observations were made: no anomalies were reported, identical spectra were found, and no strong absorption was observed. For example, we can clearly observe in all the spectra, the band at 2969 cm^−1^ due to CH_2_ stretching vibration. A broad band at 3532 cm^−1^ is attributed to the stretching vibrations of the hydroxyl group in water molecules, amine and amide NH. The results of the identification tests were consistent and identical to those of the reference substance. All the analyses conducted are satisfactory and comply with standards. The crystallographic data of amoxicillin trihydrate samples obtained by XRD analyses are summarized in the following table, which aims to emphasize the effect of high and low frequency ultrasound on crystallographic data (degree of crystallinity), compared to silent conditions. It is interesting to note that they are coherent with bibliographical data [42] (See Table 5).

**Table 5:** Crystallographic data concerning the degree of crystallinity of amoxicillin crystallized under different treatments.

**Amoxicillin trihydrate crystallized**	**Intensity Crystalline Area**	**Intensity Amorphous Area**	**Degree of crystallinity (%)**
**Silent conditions**	**85216.18**	**86250.29**	**49.69 ± 0.43**
**20 kHz ultrasound**	**82001.04**	**105089.61**	**43.83 ± 0.63**
**581 kHz ultrasound**	**75682.08**	**87202.44**	**46.46 ± 0.19**

It was observed that the degree of crystallinity of amoxicillin trihydrate obtained in silent conditions or under the effect of high or low frequency ultrasound varies between 43.83 % and 49.69 %, so quite low levels. In the case of high frequency (581 kHz), this degree (46.46 %) is close to that obtained in the case of crystallization in silent conditions (49.69 %). This means that the high frequency led to the appearance of amorphous particles at a low rate compared to low frequency. In a recent review of amorphous pharmaceutical solids by Edina Vranic [43], it was reported that amorphous substances are an important class of pharmaceutical materials with distinct physical and chemical properties. They can form intentionally or unintentionally during normal pharmaceutical manufacturing operations. Pathways to the amorphous state include molecular quenching of melts, rapid precipitation by antisolvent addition, freeze-drying, spray-drying, and the introduction of impurities. In our case, the low crystallinity degrees and the amorphous particles presence are probably due to the rapid precipitation, by the physical action of ultrasound applied to the amoxicillin trihydrate particles, leading to more or less significant fragmentation of the particles formed depending on the frequency used. Our results are in perfect agreement with those reported by [44], i.e. crystallinity is not a constant characteristic but can be easily modified by physical or thermal treatments. During the crystallization process, this is the only explanation that can be formulated in our case, since crystallization of amoxicillin trihydrate was followed by the application of the same filtering and drying protocol (time and temperature).

#### 3.2.3 Effect of sonication time on crystals morphology at different frequencies

**Fig. 12:**
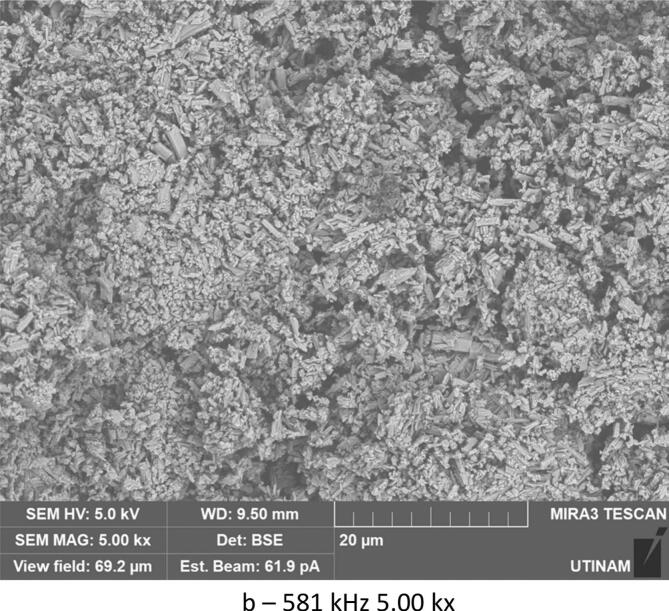

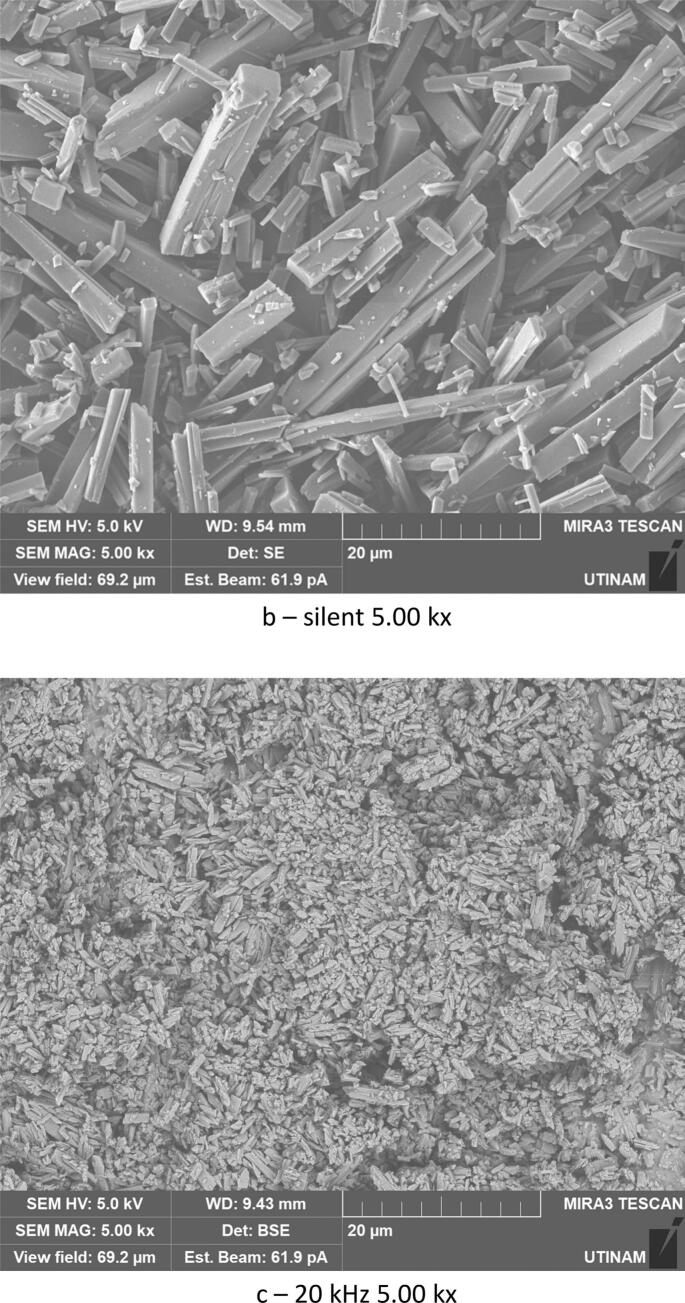
SEM analysis of crystals obtained under different treatment with the same magnification 5.00 kx after 10 min sonication/stirring (tests were carried out using the same concentration of 0.040 g/mL in sodium amoxicillin at pH 4.5 and using a power of around 7.25 W for the ultrasound treatment and a stirring speed of 100 rpm for silent conditions).

**Fig. 13:**
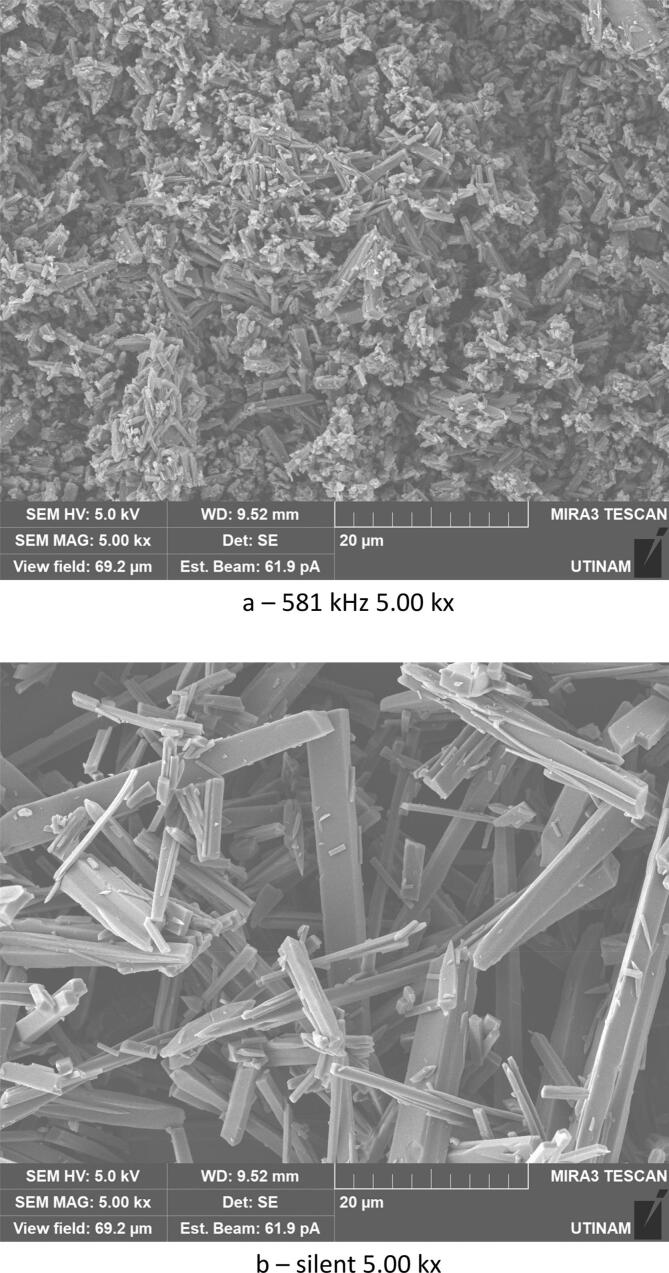

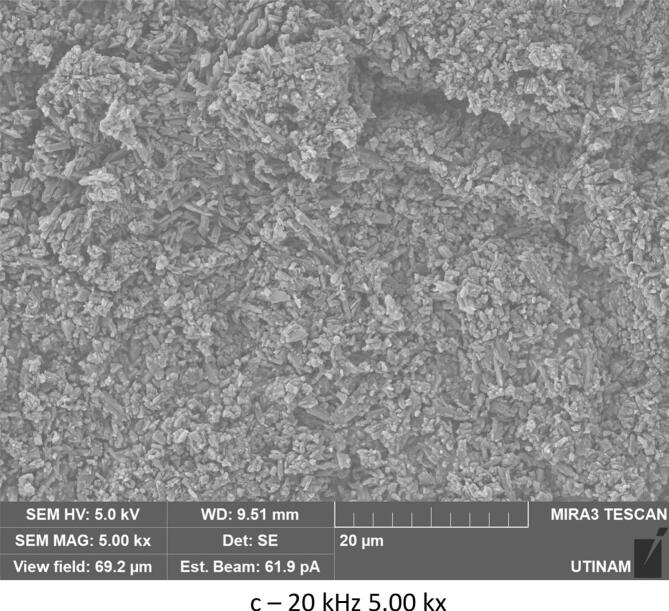
SEM analysis of crystals obtained under different treatments with the same magnification 5.00 kx after 30 min sonication/stirring (tests were carried out using the same concentration of 0.040 g/mL in sodium amoxicillin at pH 4.5 and using a power of around 7.25 W for the ultrasound treatment and a stirring speed of 100 rpm for silent conditions).

By varying sonication time, it was found that with 30 min under low frequency ultrasound (20 kHz), smaller crystals were formed compared to those obtained under shorter sonication times (10 min). The crystallized amoxicillin trihydrate particles have an average width of about 0.53 μm after 10 min (Fig. 12), and when extended to 30 min, sonication reduces the average size to 0.41 μm (Fig. 13). A similar effect was obtained in silent conditions: long stirring leads to reduction in particle size. The particles of amoxicillin trihydrate crystallized have an average width of around 1.46 µm under the effect of 10 min of stirring, while extended to 30 min reduced the average size to 1.23 µm.

According to Narducci et al., under short sonication times, it is possible that the solute does not receive the required driving force without a sufficient duration, and therefore a larger size is obtained. While crystals generated under silent conditions are irregularly shaped and of various sizes, but prolonged sonication times improve mixing, exposing the majority of crystals to cavitation bubbles and thus preventing unwanted crystal growth leading to smaller sizes [45] The same results were obtained with various organic compounds [[46], [47], [48], [49]]. In the case of high frequency 581 kHz, increasing sonication times led to an inverse effect. An increase in size was noted, with particle width increasing from 0.279 μm to 0.530 μm.

**Fig. 14:**
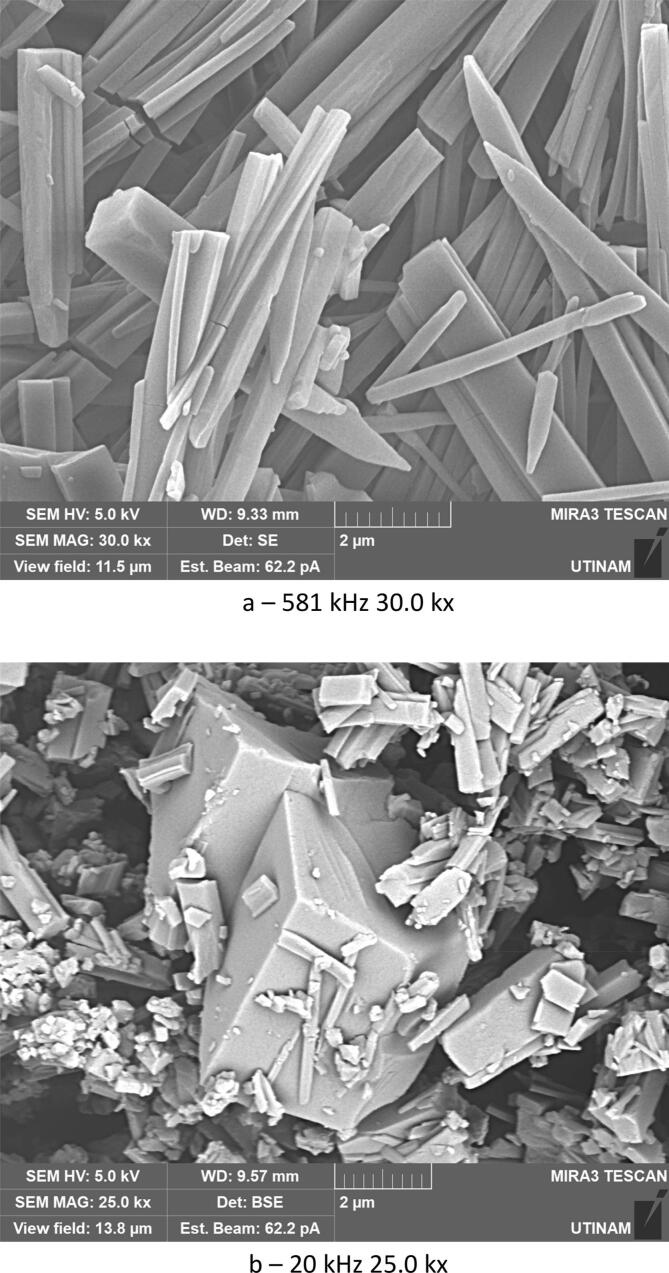
High magnifications of SEM pictures of amoxicillin trihydrate after 30 min sonication at 581 kHz and 20 kHz.

According to the results obtained in terms of frequency and duration of sonication, it appears that application of ultrasound with different frequencies leads to fragmentation and deformation of crystallized trihydrated amoxicillin particles, as it shown in Fig. 14, leading to different degrees of deformation and reduction in size. This is probably due to the physical effects caused by acoustic cavitation due to application of ultrasound in a heterogeneous medium (solid liquid). Some researchers have reported that when the solid particles in the mixture are smaller than the resonance size of the bubble (expected to be around 100 µm at 20 kHz [50], decreasing to a few µm at high frequency), the shock wave generated by acoustic cavitation causes interparticle collisions [51,52]. This is the case for all of our conditions. According to Zeiger et al., shock waves interact directly with the solid particles, causing sonofragmentation of the solid particles [53]. The same conclusion was drawn by Suslick et al., in a related study on aspirin slurry under sonication after suggesting four possible mechanisms of particle breakage causing sonofragmentation including interparticle collision, particle-horn collision, particle–wall collision, and direct interaction between particles and shock waves (i.e. sonofragmentation) [54].

Trihydrate amoxicillin crystallized in orthorhombic form in both cases (under silent and ultrasonic conditions), but with the application of ultrasound, a new deformed form of the sharp-ended orthorhombic form appeared.

### 3.3 Overview of the crystallization behavior

**Fig. 15:**
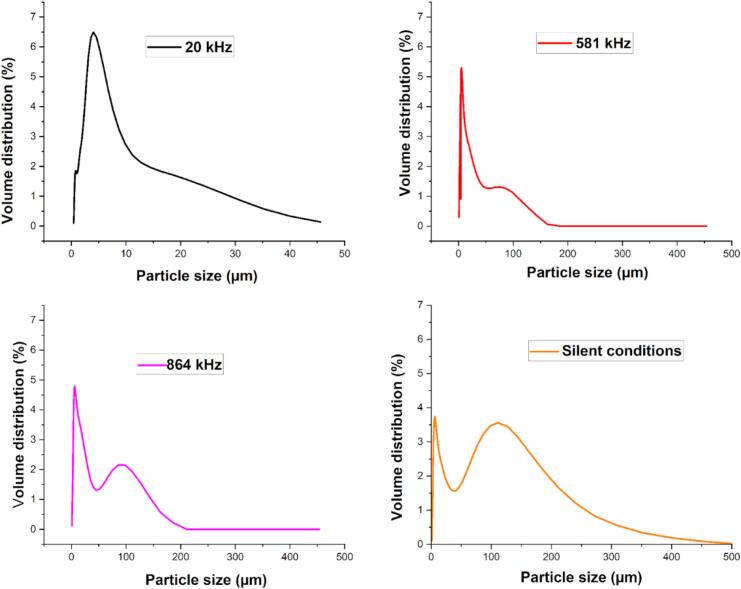
Example of size distribution of amoxicillin trihydrate obtained in liquid particle size distribution using Malvern Mastersizer 3000.

To try to understand the crystallization of amoxicillin behavior, a study of the morphology and distribution of formed solid particles was undertaken (after completion of all stages of the process of transformation of sodium amoxicillin into amoxicillin trihydrate), as well as of particles formed in suspension, before the filtering and drying steps. The behavior of crystallized particles of amoxicillin trihydrate in powder or in suspension form (without passing through filtering and drying) was studied and illustrated by examining SEM and XRD photos for the first case and using the laser particle size diffraction technique for the second case. We highlighted the effect of temperature, the effect of pH, and also the sonication parameter applied on the morphology and the size of the formed particles. The size of crystal particles in different suspensions from different crystallization experiments was measured and shown in Table 6, which summarizes all results. The particle distribution diagram of crystals formed without or with the application of low or high frequency ultrasound (20, 581, and 864 kHz) is illustrated in Fig. 15.

**Table 6:** Particle size data from different tests (in suspension form).

**Sample number**	**Frequency**	**Final crystallization pH**	**Particle size range (µm)**	**% of dominant particle size (µm)**
1	Without ultrasound	4.5 Complete crystallization	0.7–250	5.92 with 4.91 % 86 with 1.93 %
2	Without ultrasound	3.54	1.8–500	4.03 with 1.29 % 127 with 5.28 % 31 with 4.37 %
3	Without ultrasound	2.73	0.9–800	5.21 with 4.41 % 310 with 2.68 %
4	Without ultrasound	5.24	1–650	6.72 with 3.69 % 98.1 with 3.47 %
5	Without ultrasound	4.53	0.7–200	8.68 with 4.44 % 98.1 with 1.38 %
6	20 kHz 7.25 W	5.44	0.6–90	5.92 with 5.40 %
7	20 kHz 7.25 W	7.21	1–200	9.80 with 4.90 %
8	20 kHz 29.5 W	7.36	0.4–60	4.30 with 6.30 % 1.45 with2.30 % 11.2 with 2.37 %
9	581 kHz 29.5 W	7.51	1–200	5.25 with 5.31 %
10	864 kHz 29.5 W	7.57	1–250	5.92 with 4.79 % 86.4 with 2.16 %

It can be observed from Table 6 that the crystallization pH plays a decisive role with regard to the size of the particles formed. When the crystallization is complete (pH around 4.5 considered as optimal pH), the size (i.e. the width in µm) of the particles formed varies between 0 and 250 µm. For a pH higher than the optimal pH, particles tend to crystallize with a width spanning a larger range varying between 1 and 650 µm while at a more acidic pH, for example at pH = 2.73, very varied wide size particles were formed with an oscillating width varying between 0.9 and 800 µm.

With respect to the size distribution of crystallized particles in solid form using SEM analysis and ImageJ and origin pro* software*, the data collected from the different treatments are illustrated in the following Figure.

**Fig. 16:**
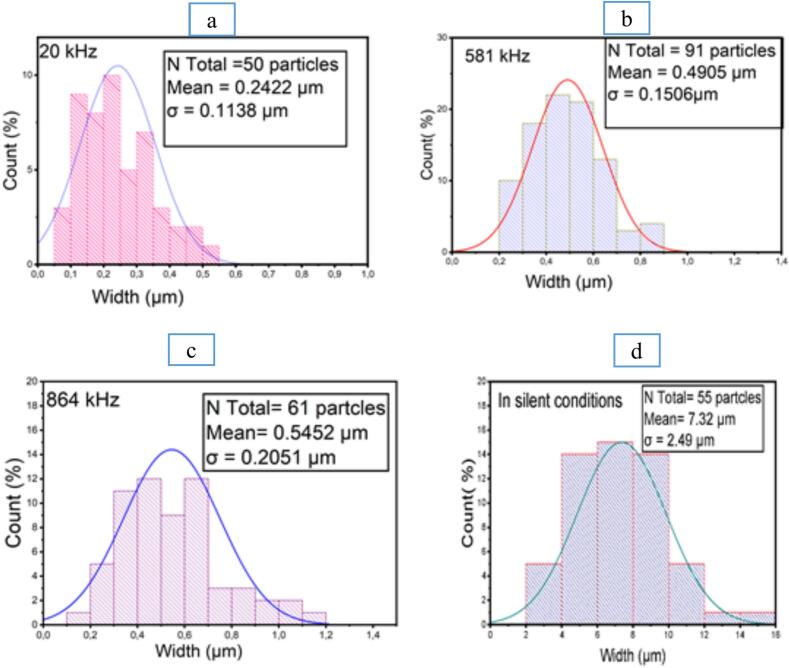
Effect of ultrasound on particle size distribution of amoxicillin trihydrate (in solid form): (a) 20 kHz, (b) 581 kHz, (c) 864 kHz (at pH = 4.5, under 30 min sonication and with 29.5 W), (d) without ultrasound (at pH = 4.43, 22 °C, 30 min and stirring at 100 rpm).

The study of granulometric distribution of crystallized amoxicillin trihydrate under different treatments performed using Image-J software in Fig. 16, indicates that it is a Gaussian distribution, but with different sizes ranging from 2 to 16 μm in silent conditions. In the case of ultrasonically crystallized molecules, their sizes gravitate around 0.2–1 μm for those resulting from crystallization under the effect of high frequencies at 581 kHz and of the order of 0.2–1.2 µm for those obtained under irradiation at 864 kHz. However, for those obtained under the effect of low frequencies at 20 kHz, amoxicillin trihydrate was crystallized with a size varying between 0.07 and 0.6 μm.

Applying three frequencies (20, 581, and 864 kHz) and following the evolution of crystallization of amoxicillin trihydrate up to a pH of around 7, the results showed that frequency has a considerable effect on the size of the particles formed. Indeed, particles of the order of 0.4–60 µm were obtained by applying low frequency 20 kHz, while it was found that when using high frequency 864 kHz particles, sizes of the order of 1–250 µm were formed. It was also noted that the ultrasound power applied has a significant effect on the size of the particles formed: application of 20 kHz ultrasound at a power of 29.5 W leads to a finer powder compared to particles obtained with a power of 7.25 W. In the case of high power, width varies between 0.4 and 60 µm with a maximum distribution around 4 µm, while for low power, diameter varies between 1 and 200 µm with a maximum distribution around 9.80 µm.

**Fig. 17:**
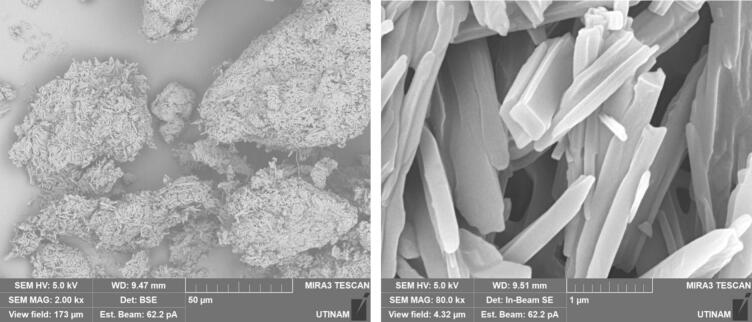
SEM analysis of crystals obtained at pH = 4.5 under 20 kHz.

**Fig. 18:**
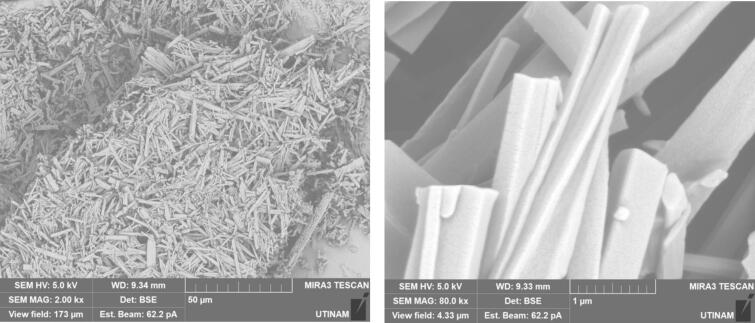
SEM analysis of crystals obtained at pH = 4.5 under 864 kHz.

The effect of pH on the behavior of the crystallized particles was also evaluated by stopping the reaction of transformation of sodium amoxicillin into amoxicillin trihydrate before completion of the reaction at pH = 3 (very few crystals) and close to the optimum around pH 4.5 at 20 kHz (Fig. 17) or 864 kHz (Fig. 18). For a given frequency, SEM images show that pH has little impact on morphology, but rather, on the contrary, on the size of crystallized particles in terms of length and width. Considerable differences may be noticed when comparing two frequencies.

Finally, the crystallization kinetics of amoxicillin trihydrate were monitored, under different treatments without ultrasound or under the effect of low or high frequencies. As weighing is not fast enough, UV–Vis spectrophotometry was used by taking volumes of the reaction medium and diluent and reading the optical density at the wavelength of 272 nm using 1-cm quartz cells, and subsequently determining the concentration by referring to a calibration curve of the initially prepared amoxicillin trihydrate and calculating the crystallization yield.

**Fig. 19:**
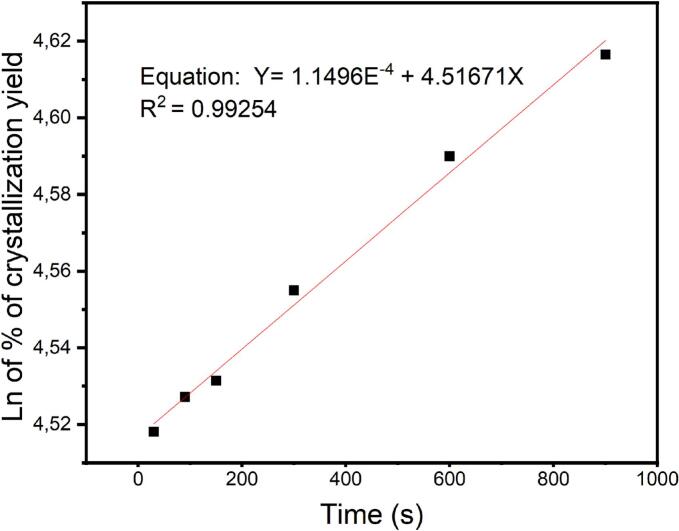
Kinetics of amoxicillin trihydrate precipitation crystallization reaction at low frequency (20 kHz).

Moreover, ultrasound use leads to a complete crystallization in less than 60 s. As a first result, we observed that nucleation is sensitive to sonication and that it was accelerated by the use of ultrasound, leading to a shorter induction time. Indeed, induction times observed in silent conditions are longer than those obtained using ultrasound (high and low frequencies). At low frequency (20 kHz), 7.0 ± 1.0 s were sufficient to make the first crystals appear, while in the case of high frequencies about 20.0 ± 1.0 s were required for 864 kHz, and 15.0 ± 2.0 s for 581 kHz. These results agree with several works carried out whether on organic or mineral molecule crystallization [14,31,55,56]. If we examine more closely the effect of 20 kHz by plotting the logarithm rate of crystallization vs sonication time (Fig. 19), reaction kinetics appear to be of the first-order with the reaction constant rate (k) observed at 0.0001 s^−1^.

### 3.4 Antibacterial activity of amoxicillin trihydrate crystallized by silent and sonochemical processes

**Fig. 20:**
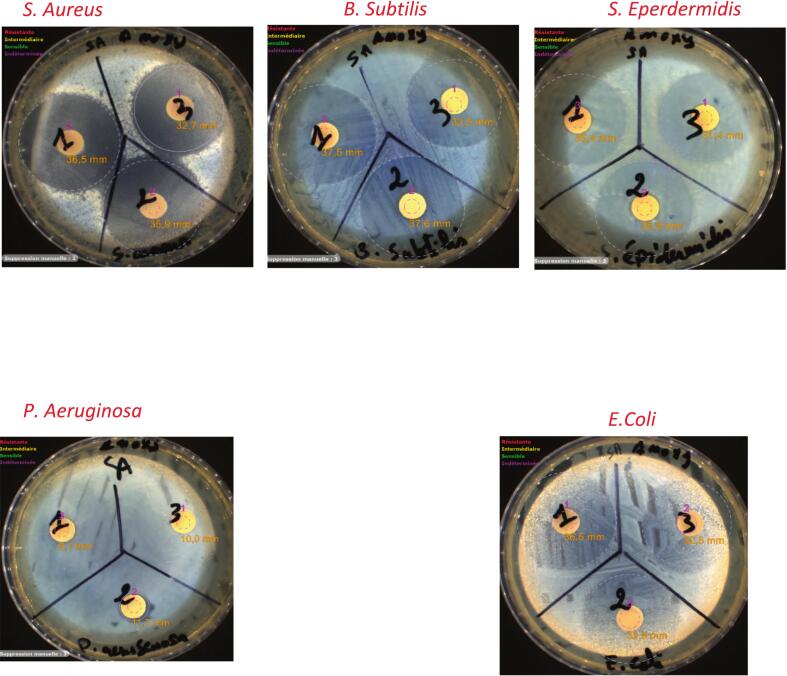
The inhibition zone against *S. Aureus, B. Subtilis, S. Eperdermidis, P. Aeruginosa,* and *E.Coli,* of amoxicillin trihydrate samples crystallized in the following manner: (1) in silent conditions, (2) under 20 kHz (3) under 581 kHz.

Antibacterial activities of amoxicillin trihydrate were tested against G-negative and G-positive bacteria using the disk diffusion assays. The results grouped in Fig. 20 and Table 7 showed that all the crystallized amoxicillin samples tested, with or without ultrasound, have the same antibacterial activities against the chosen microorganisms but not of the same intensity. Concerning the diameter of the inhibition zone, we noticed that the samples crystallized under the effect of low frequency ultrasound 20 kHz present larger diameters compared to the samples crystallized under high frequency ultrasound (581 kHz). In silent conditions, the largest diameter was observed against *B. Subtili* (37.6 mm).

**Table 7:** Antibacterial activities of Amoxicillin trihydrate against pathogenic microorganisms.

**Microorganism**	**Zone of inhibition (mm)**		
	**Amoxicillin trihydrate (silent)**	**Amoxicillin trihydrate (20 kHz)**	**Amoxicillin trihydrate (581 kHz)**
***S. Aureus***	**36.5 ± 0.1**	**35.9 ± 0.2**	**32.7 ± 0.3**
***B. Subtilis***	**37.5 ± 0.1**	**37.6 ± 0.1**	**33.5 ± 0.2**
***S. Eperdermidis***	**35.4 ± 0.3**	**35.8 ± 0.2**	**31.4 ± 0.2**
***P. Aeruginosa***	**9.1 ± 0.2**	**11.7 ± 0.1**	**10.0 ± 0.2**
***E.Coli***	**36.5 ± 0.1**	**33.9 ± 0.1**	**32.5 ± 0.2**

The highest antibacterial ability of amoxicillin trihydrate crystallized at low frequency (20 kHz) against *B. Subtilis****,*** *S. Eperdermidis,* and *P. Aeruginosa* can be related to their very small particle size, 10 times smaller than that precipitated in silent conditions. It was also found that particle size may play an important role in the antimicrobial activity.

As particle size decreases, the ratio of surface to volume of the particle increases. Smaller particles with larger surface-to-volume ratios have greater antibacterial activity since the antibacterial properties are related to the total surface area of the microparticles. As reported by Cho et al., antibacterial activity decreases with increasing particle size [57,58]. This result may be due to strong particle scattering for the smallest sizes. Their well-developed surface causes them to interact with microorganisms through easier adhesion to the cell wall of the micro-organism, causing its destruction and death [59].

## 4 Conclusion

The process of transforming sodium amoxicillin into amoxicillin trihydrate by crystallization or sonocrystallization by adjusting pH is both a feasible and rapid process, leading to products that meet the standards required by the European and American (United States) pharmacopeias. The important findings resulting from this work considered as the main conclusions, are summarized as follows:

- Ultrasound has a beneficial effect on crystallization of amoxicillin trihydrate by improving the yield and fineness of the powder, as well as on the kinetics of the reaction with regard to the nucleation time. This is particularly the case with low frequency 20 kHz, where the first crystals appear at 7 s, whereas under normal conditions, this time was around 20–32 s. The best yield obtained by applying low frequencies (20 kHz) was about 95 % compared to the yield obtained without ultrasound (69 %).
- Temperature, pH and duration of sonication exert a profound effect on the morphology and size of product crystals. When low frequency is used, particle size was found to range from 0.4 to 60 µm under sonication and from 0.7 to 250 µm in silent conditions. However, the heat generation by ultrasound shifts the temperature range of the study to higher values (30–34 °C). Although this can be considered closer to real processing conditions, future works should involve conducting ultrasound experiments with better temperature control (including the optimal temperature found 7 °C in silent conditions).
- The key factor limiting the sonocrystallization process is the pH. It was found that when pH = 4 is exceeded, yield decreases considerably, whereas for a pH ≤ 2 the crystallization yield does not exceed 10 %.
- Ultrasound modifies the size and morphology of crystals, and even slightly increases the therapeutic effectiveness of crystalline amoxicillin as shown by antibacterial activity tests.

## CRediT authorship contribution statement

**Aicha Ladaidi:** Writing – original draft, Methodology, Investigation, Conceptualization. **Loïc Hallez:** Formal analysis, Conceptualization. **Isabelle Pochard:** Methodology, Formal analysis. **Nicolas Rouge:** Methodology, Investigation, Formal analysis. **Jean-Yves Hihn:** Writing – review & editing, Writing – original draft, Supervision, Conceptualization.

## Declaration of competing interest

The authors declare that they have no known competing financial interests or personal relationships that could have appeared to influence the work reported in this paper.
