# Supplementary material for: The effect of ultrasound on the crystallization-precipitation process of transforming sodium amoxicillin into amoxicillin trihydrate
Source: Ultrason Sonochem. 2025 Sep 26;122:107590. doi: 10.1016/j.ultsonch.2025.107590 (PMC12516560; doi:10.1016/j.ultsonch.2025.107590)
Supplement: Supplementary Data 1 [file mmc1.docx]

SM1: UV/Vis calibration curve of amoxicillin trihydrate (reference substance 94.86%)


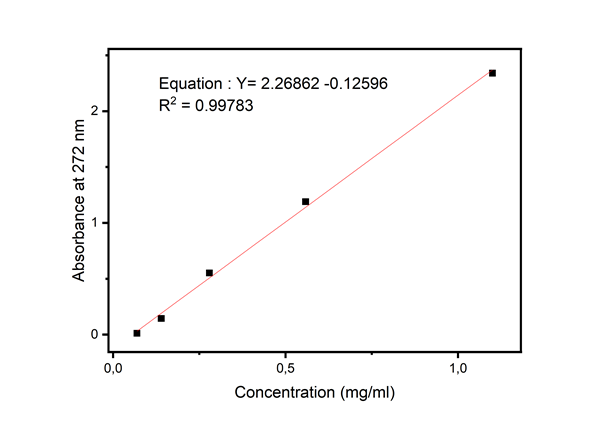


SM2: The crystallization study of amoxicillin trihydrate was based on the solubility data reported for amoxicillin trihydrate at 37°C in the literature by A. Tsuji et al [33] (Table 1)

| pH | Solubility (mg/mL) |
| --- | --- |
| 1.72 | 72.56 |
| 1.94 | 32.29 |
| 2.42 | 14.43 |
| 2.89 | 9.9 |
| 4.05 | 5.49 |
| 4.16 | 5.62 |
| 4.27 | 5.2 |
| 4.37 | 5.33 |
| 4.48 | 5.45 |
| 4.71 | 5.45 |
| 4.97 | 5.41 |
| 5.98 | 5.66 |
| 6.5 | 6.33 |
| 6.87 | 8.97 |
| 7.19 | 10.32 |
| 7.19 | 10.32 |
| 7.64 | 19.29 |

95 % of crystals formed and precipitated, not dissolved. To calculate the amount used, use the following formula:

To determine the concentration of crystallized amoxicillin over time, use the equation for the calibration curve: Absorbance = 2.2686.X -0.126

Found amoxicillin concentration (mg/mL) = (Found optical density +0.126) / 2.2686

Actual concentration (mg/mL) = Found amoxicillin concentration (mg/ml) * dilution

Found mass of amoxicillin = Actual concentration (mg/mL * total volume)

Crystallization yield = (mass of precipitated amoxicillin / the initial mass of dissolved sodium amoxicillin)*100)
